# Combination of Decitabine and Entinostat Synergistically Inhibits Urothelial Bladder Cancer Cells via Activation of FoxO1

**DOI:** 10.3390/cancers12020337

**Published:** 2020-02-03

**Authors:** Chenyin Wang, Alexandra Hamacher, Patrick Petzsch, Karl Köhrer, Günter Niegisch, Michèle J. Hoffmann, Wolfgang A. Schulz, Matthias U. Kassack

**Affiliations:** 1 Institute of Pharmaceutical and Medicinal Chemistry, Heinrich-Heine-University Duesseldorf, 40225 Duesseldorf, Germany; chenyin.wang@hhu.de (C.W.); alexandra.hamacher@hhu.de (A.H.); 2 Biological and Medical Research Center (BMFZ), Medical Faculty, Heinrich-Heine-University Duesseldorf, 40225 Duesseldorf, Germany; patrick.petzsch@hhu.de (P.P.); koehrer@hhu.de (K.K.); 3 Department of Urology, Medical Faculty, Heinrich-Heine-University Duesseldorf, 40225 Duesseldorf, Germany; guenter.niegisch@med.uni-duesseldorf.de (G.N.); michele.hoffmann@hhu.de (M.J.H.); wolfgang.schulz@hhu.de (W.A.S.)

**Keywords:** bladder cancer, cisplatin, DNA methyltransferase inhibitor, drug combination study, histone deacetylase inhibitor, forkhead box class O1

## Abstract

Occurrence of cisplatin-resistance in bladder cancer is frequent and results in disease progression. Thus, novel therapeutic approaches are a high medical need for patients suffering from chemotherapy failure. The purpose of this study was to test the combination of the DNA methyltransferase inhibitor decitabine (DAC) with the histone deacetylase inhibitor entinostat (ENT) in bladder cancer cells with different platinum sensitivities: J82, cisplatin-resistant J82CisR, and RT-112. Intermittent treatment of J82 cells with cisplatin resulted in the six-fold more cisplatin-resistant cell line J82CisR. Combinations of DAC and/or ENT plus cisplatin could not reverse chemoresistance. However, the combination of DAC and ENT acted cytotoxic in a highly synergistic manner as shown by Chou-Talalay analysis via induction of apoptosis and cell cycle arrest. Importantly, this effect was cancer cell-selective as no synergism was found for the combination in the non-cancerous urothelial cell line HBLAK. Expression analysis indicated that epigenetic treatment led to up-regulation of forkhead box class O1 (FoxO1) and further activated proapoptotic Bim and the cell cycle regulator p21 and reduced expression of survivin in J82CisR. In conclusion, the combination of DAC and ENT is highly synergistic and has a promising potential for therapy of bladder cancer, particularly in cases with platinum resistance.

## 1. Introduction

Bladder cancer is the 6th most common cancer in the United States with an estimated 80,470 new cases and 17,670 deaths in 2019 [1]. Among the different types of bladder cancer, urothelial carcinoma is the most common histological subtype, which can be further divided into superficial and muscle-invasive, based on the depth of the tumor invasion into the wall of the bladder [2]. Although only 30% newly diagnosed bladder cancer cases are advanced, another 15–30% of high-grade superficial bladder cancers develop into muscle-invasive carcinomas with poor long-term survival [3]. Generally, the treatment options of bladder cancer include surgery, radiation therapy, and cisplatin-based chemotherapy [4]. Cisplatin enters cells through passive diffusion and mediated by membrane transporters [5]. The cytotoxic effect is then achieved by the formation of DNA adducts leading to apoptosis and cell cycle arrest [6]. Although cisplatin is initially effective in most cases, acquisition of cisplatin resistance results in cancer relapse, significantly reducing its clinical usefulness. Development of cisplatin resistance is multifactorial, including pre-target, on-target, and post-target mechanisms, corresponding to diminished intracellular drug accumulation, increased rates of DNA damage repair, and defects in apoptotic signal transduction pathways which are normally activated in response to DNA damage, respectively [7]. Collectively, these processes will then inhibit apoptosis and subsequently lead to drug resistance [7]. Therefore, it is important to search for novel strategies in bladder cancer treatment, also for those patients ineligible for platinum-based therapy. One such approach is epigenetic modulation.

Aberrant epigenetic modifications play a significant role in the genesis, progression, and chemoresistance of cancer, including bladder cancer [8,9]. One fundamental epigenetic mechanism is DNA methyltransferase (DNMT)-mediated DNA methylation occurring by addition of a methyl group to carbon 5 of cytosine. In cancers, aberrant DNA methylation affects promoter regions (CpG islands) with subsequent inhibition of transcription, particularly in tumor suppressor genes [10]. DNMT inhibitors (DNMTi’s) such as 5-azacytidine or decitabine have been approved for treatment of acute myeloid leukemia (AML) and myelodysplastic syndrome (MDS) and act in part by reactivating genes silenced by abnormal promotor hypermethylation [11].

In addition to DNA methylation, histone modifications are also a crucial mechanism in transcription regulation. The acetylation or deacetylation of lysine residues on histones regulates chromatin structure and gene transcription. These processes are mediated by two enzyme families known as histone acetyltransferases (HATs) and histone deacetylases (HDACs) [12]. Acetylation of histones is in general correlated with active gene transcription, whereas HDAC-mediated hypoacetylation is associated with transcriptional repression [13]. Depending on sequence similarity, zinc-dependent HDACs can be grouped into class I (HDAC1, 2, 3, and 8), class II (IIa: HDAC4, 5, 7, 9; IIb: 6 and 10), and class IV (HDAC11) [14]. In many types of cancer, including bladder cancer, increased expression levels of HDACs have been observed and frequently relate to poor clinical prognosis [15,16]. As a result, the aim of targeting HDACs has prompted the development of HDAC inhibitors (HDACi’s). During the last years, different types of HDACi’s, broad-spectrum (pan inhibitors) or selective HDACi’s, have been developed and evaluated for treatment of solid tumors and different types of lymphomas [17]. Four HDACi’s are US-FDA approved for the treatment of various lymphomas: vorinostat (cutaneous T-cell lymphoma (CTCL)), romidepsin (CTCL), belinostat (peripheral T-cell lymphoma (PTCL)), and panobinostat (multiple myeloma) [17]. The HDACi chidamide is approved by the Chinese FDA for PTCL [18]. Vorinostat, belinostat, and panobinostat are pan HDACi’s, whereas romidepsin and chidamide are class I-selective HDACi’s [11]. Recently, our group has contributed several highly potent and subtype-selective HDACi’s leading to a complete reversal of platinum resistance in ovarian and head-neck cancers [19,20,21,22]. In a recent study, we could moreover demonstrate that class I HDAC inhibition by entinostat was superior to pan-HDAC inhibition in high-grade serous ovarian carcinomas in reversing chemoresistance against cisplatin [21]. Notably, class I HDACs show increased expression in many cancer cells and promote tumor cell proliferation [23]. This fact and the results from our study in ovarian cancer cells suggest that selective class I HDAC inhibition is more promising than pan-HDAC inhibition and avoids side effects due to lack of selectivity [21]. Another study of our groups rules out a significant role of class IIa HDACs in bladder cancer [24]. Taken together, these data suggest that it should be worthwhile trying class I HDACi’s for chemoresistance reversal in bladder cancer. Thus, we decided to use entinostat, a class I selective benzamide currently undergoing clinical studies in several cancers, including bladder cancer [25,26]. In addition, based on the fact that DNMTi’s and HDACi’s both serve to regulate transcriptional activity, combining these two types of inhibitors might provide a clinical strategy for bladder cancer treatment, especially for patients who have already developed cisplatin resistance [11]. Thus, the aim of this study was: first, to establish-starting from the J82 bladder cancer cell line-a cisplatin-resistant subline (J82CisR) according to clinically relevant protocols previously published [27]; second, to investigate the efficacy of the DNMTi decitabine (DAC) and HDACi entinostat (ENT) alone and in combination to inhibit the growth of platinum-sensitive (J82) and platinum-resistant (J82CisR, RT-112) bladder cancer cells.

## 2. Results

### 2.1. Combining DNMTi and HDACi Induces Synergistic Cytotoxicity in Urothelial Bladder Cancer Cell Lines

**Figure 1:**
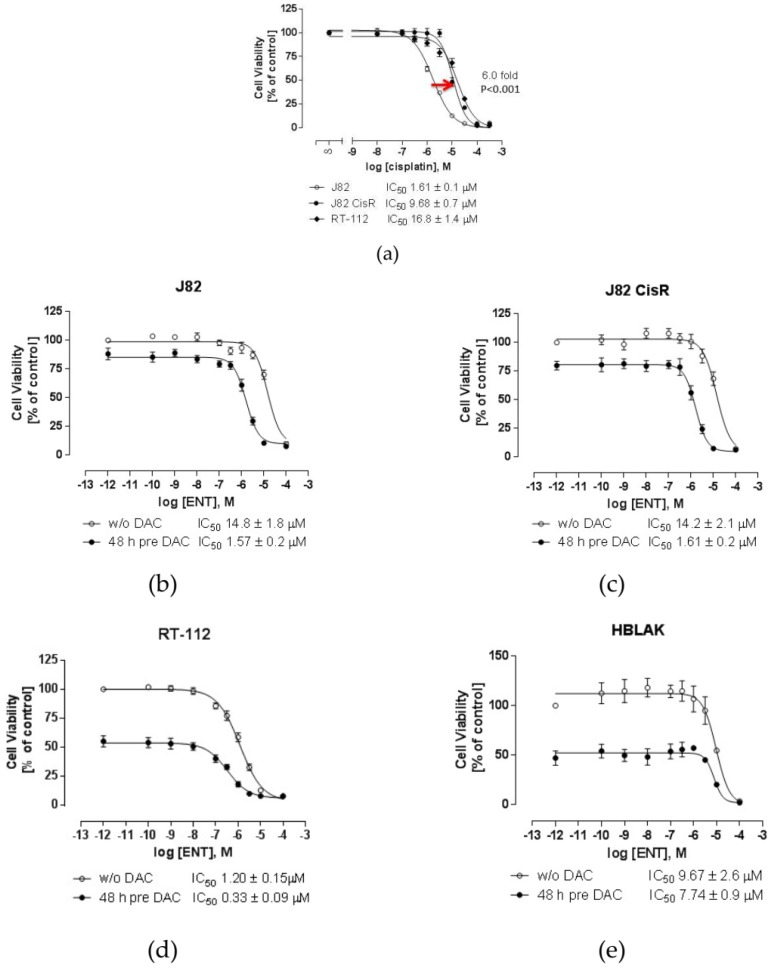
Generation of the cisplatin-resistant cell line J82CisR and combination treatment with DAC and ENT. (**a**) Thirty-four cycles of weekly exposure of J82 with cisplatin for 6 hours resulted in 6-fold (*p* < 0.001) increase in IC_50_ [inhibitory concentration 50%] of cisplatin in J82CisR as indicated by the red arrow. IC_50_ of cisplatin in J82: 1.61 µM; IC_50_ of cisplatin in J82CisR: 9.68 µM. Data shown are mean ± SEM, *n* = 3. (**b**) Forty-eight hours pre-incubation with DAC (1 µM) significantly enhanced the cytotoxicity of ENT in J82 cell line by decreasing IC_50_ from 14.8 µM to 1.57 µM with a shift factor of 9.4. (**c**) Pre-incubation with DAC (1 µM) decreased IC_50_ of ENT from 14.2 µM to 1.61 µM in J82CisR. (**d**) Pre-incubation with DAC (0.1 µM) increased the cytotoxic effect of ENT in RT-112 as shown by a shift factor of 3.6. (**e**) Pre-incubation of DAC (1 µM) did not significantly increase the cytotoxic effect of ENT in the normal human bladder cell line HBLAK. “% of control” on the y-axis means: % of untreated cells.

The cisplatin-sensitive J82 cell line (IC_50_ [inhibitory concentration 50%]: 1.61 µM) was intermittently exposed to cisplatin (1.6 µM, reflecting the IC_50_ value of J82) for 6 h. After several treatment cycles, the IC_50_ increased from 1.61 µM (J82) to 9.68 µM (J82CisR) corresponding to a resistance factor of 6 (Figure 1a). Notably, no further cisplatin treatments were needed to maintain the increased IC_50_ of cisplatin in J82CisR indicating a stable resistance. Furthermore, RT-112 cells were used displaying native cisplatin resistance (IC_50_: 16.8 µM, Figure 1a). Thus, in total, three urothelial bladder cancer cell lines were used as in vitro models.

The cytotoxicity of the DNMTi DAC and the class I HDACi ENT was determined using 3-(4,5-di**m**ethyl**t**hiazol-2-yl)-2,5-diphenyl**t**etrazolium bromide (MTT) assay. In J82 and J82CisR cell lines, DAC showed weak cytotoxicity with IC_50_ values of 30.5 and 28.2 µM, respectively (Table 1; Figure S1). In contrast, RT-112 cells were dramatically more sensitive to DAC showing an IC_50_ value of 0.18 µM. Similarly, the HDACi ENT was more potent in RT-112 compared with J82 and J82CisR cell lines, with IC_50_ values of 3.41, 14.3, and 15.6 µM, respectively.

Next, the combination of DAC with cisplatin or ENT with cisplatin was tested. Similar to our previous studies with HDACi and considering the time-dependent effects of DNMTi and HDACi, DAC or ENT were incubated with the respective cell lines 48 h prior to addition of cisplatin [19,21]. The resulting concentration-effect curves of cisplatin are shown in Figure S2 for all three cell lines. In contrast to our previous studies in head-neck or ovarian cancer cells [19,21], ENT did not completely but only partially reverse cisplatin resistance in J82CisR. While the resistance factor between J82 and J82CisR is 6, the partial reversal of cisplatin resistance gave a shift factor of only 1.5 (Figure S2b).

**Figure 2:**
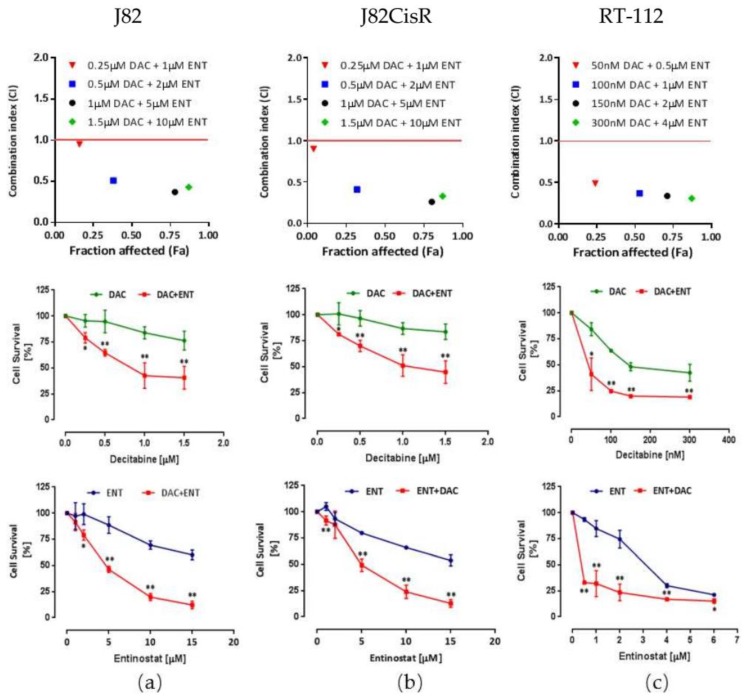
Combination of DAC and ENT elicits synergistic cytotoxic effects in urothelial bladder cancer cells. (**a**) J82; (**b**) J82CisR; (**c**) RT-112 cells. Upper panel: cells were treated for 48 h with DAC, followed by 72 h of ENT, respectively; x axis indicates fraction of cells affected (Fa); y axis indicates combination index (CI). Synergistic effect is defined by a CI below the red line (CI < 1). Middle and bottom panels: percentage of cell survival after treatment with DAC or ENT alone or in combination. Data shown are the mean ± SD of at least three independent experiments. ** *p* < 0.01, * *p* < 0.05, *t*-test, combination treatment compared with single treatments.

Thus, we next investigated the dual combination of DAC and ENT (without cisplatin) for synergistic effects. Exploratory experiments investigating different sequences of the two compounds revealed that coincubation or preincubation of an HDACi prior to DAC treatment resulted in an additive but not synergistic effect. Therefore, and given the fact that DNA methylation can even influence the expression of HDACs [10], DAC was incubated prior to the treatment of ENT. Our results show that the cytotoxic effect of ENT was significantly enhanced by pretreatment with DAC in all tested cell lines. In the presence of low-dose DAC (1 µM in J82 and J82CisR, 0.1 µM in RT-112), the IC_50_ values of ENT decreased 9.4-, 8.8-, and 3.6-fold compared to the absence of DAC in J82, J82CisR, and RT-112 cell lines, respectively (Figure 1b–d). To test for cancer selectivity of these observed effects, the spontaneously immortalized normal human urothelial bladder cell line HBLAK was treated with the combination of DAC and ENT as well. In contrast to the results for the bladder cancer cell lines described above, 48 h pre-treatment with 1 µM of DAC did not significantly enhance the cytotoxicity of ENT in HBLAK cells (Figure 1e) [28]. Thus, the highly synergistic effect of DAC and ENT is selective for bladder cancer cells over non-cancerous bladder cells. The drug combination index (CI) of DAC and ENT was calculated using the Chou-Talalay drug combination study method [29]. According to the drug combination model, a synergistic effect is proven if the CI is below 1. Our results indicate that DAC and ENT act synergistically in inhibiting the growth of bladder cancer cells (Figure 2 upper panel). Furthermore, a significantly higher inhibition of cell proliferation by the drug combination compared with either agent alone is illustrated in Figure 2 middle and bottom panels.

### 2.2. Combination Treatment of DAC and ENT Induces Apoptosis in Urothelial Bladder Cancer Cells Mediated by the Activation of Caspase 3/7

**Figure 3:**
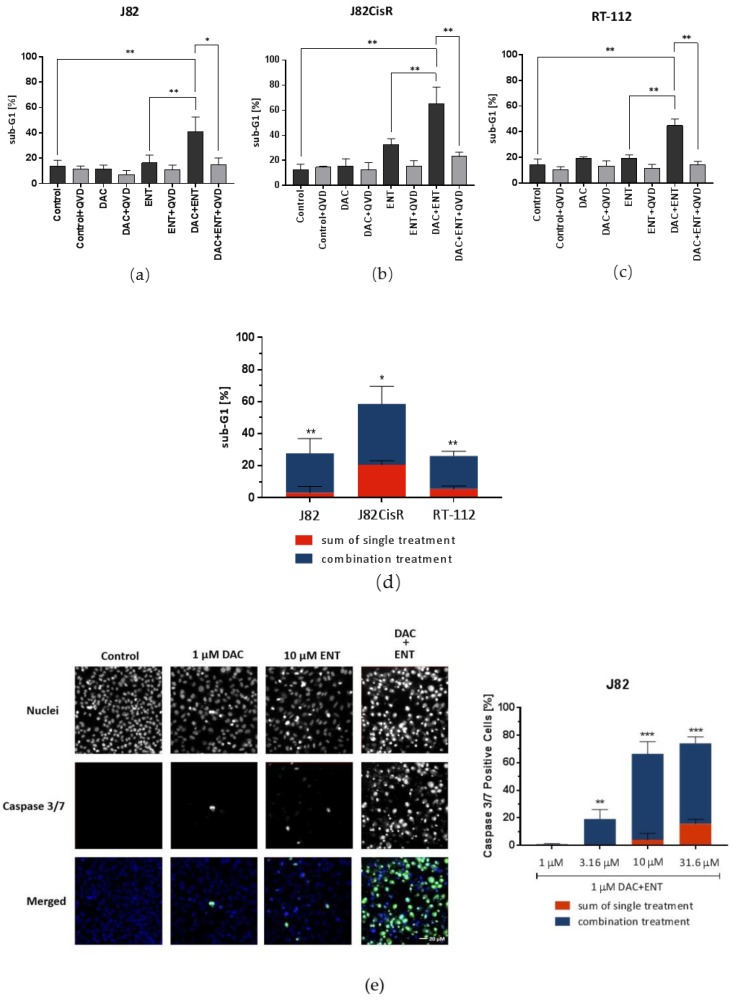

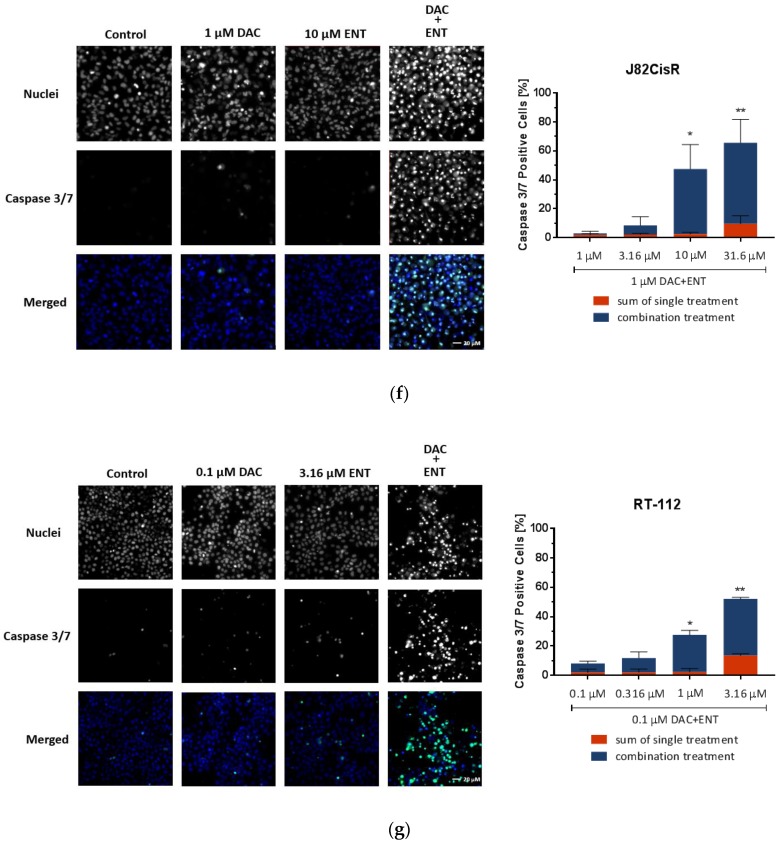
DAC and ENT acted synergistically to induce caspase 3/7-mediated apoptosis. (**a**–**c**) Combinations of DAC (1 µM in J82 and J82CisR, 0.1 µM in RT-112) and ENT (3.16 µM in J82 and J82CisR, 2 µM in RT-112) led to increased sub-G1 fractions, compared with either agent alone in J82, J82CisR, and RT-112 cell lines. In each single experiment, 12,000 cells per condition were analyzed. Medium was used as control. Data shown are the mean ± SD of at least three independent experiments. ** *p* < 0.01, * *p* < 0.05 by *t*-test. (**d**) The sums of sub-G1 signals of single treatments are shown in red bars. The differences between the combination treatment effects and the sums of single treatment effects are shown in extended blue bars (superadditive (=synergistic) part). Data shown are the mean ± SD of at least three independent experiments. ** *p* < 0.01, * *p* < 0.05 by *t*-test. (**e**–**g**) The 48 h treatment of DAC followed by 48 h ENT leads to caspase 3/7 activation in a synergistic manner in J82, J82CisR, and RT-112 cells. A minimum of 500 cells per condition were analyzed in each single experiment. Medium was used as control for untreated cells, and cisplatin (not shown) was used as positive control for caspase 3/7 activation. Cells were stained with Hoechst 33342 (blue) for cell nuclei and CellEvent Caspase-3/7 green detection reagent for the activation of caspase 3/7. Quantification of caspase 3/7 positive cells is displayed in the right panel. The sums of percentages of caspase 3/7 positive cells of single treatment are shown in red bars. The differences between the combination treatment effects and the sums of single treatment effects are shown in extended blue bars (superadditive (=synergistic) part). Data shown are the mean ± SD of at least three independent experiments. *** *p* < 0.001, ** *p* < 0.01, * *p* < 0.05 by *t*-test. Scale bars for (**e**–**g**): 20 µm.

To understand the mechanism of growth inhibition and synergistic cytotoxicity of the combination treatment of DAC and ENT shown above, apoptosis assays (sub-G1 analysis) were performed following DAC and ENT treatment either alone or combined. In agreement with the result of the drug combination study described above, the combination of DAC and ENT led to a strong increase in cells in sub-G1 phase compared to single agent treatment. Importantly, this effect was more pronounced in J82CisR cells with 65.1% sub-G1 population compared with 40.9% and 44.7% in J82 and RT-112 in the combination treatments, respectively (Figure 3a–d). The increased number of cells in sub-G1 after the combination treatment could be reversed by addition of the pan-caspase inhibitor Q-VD-OPh, indicating caspase-driven apoptosis induction. To confirm that the observed effect in sub-G1 assays was not due to other types of cell death, the activity of caspases 3 and 7 was measured using fluorescence-based high content analysis. Under treatment with either agent alone, caspase 3/7 activation was detected in only a few cells. If both drugs were combined, the number of caspase 3/7 positive cells was significantly increased to 66.6%, 49.1%, and 66.3% in J82, J82CisR, and RT-112 cells, respectively (Figure 3e–g). Taken together, these results indicate that the combination of DAC and ENT induces caspase 3/7-mediated apoptosis in all three bladder cancer cell lines in a synergistic manner.

### 2.3. DAC Combined with ENT Affects the Cell Cycle Distribution in Urothelial Bladder Cancer Cells

**Figure 4:**
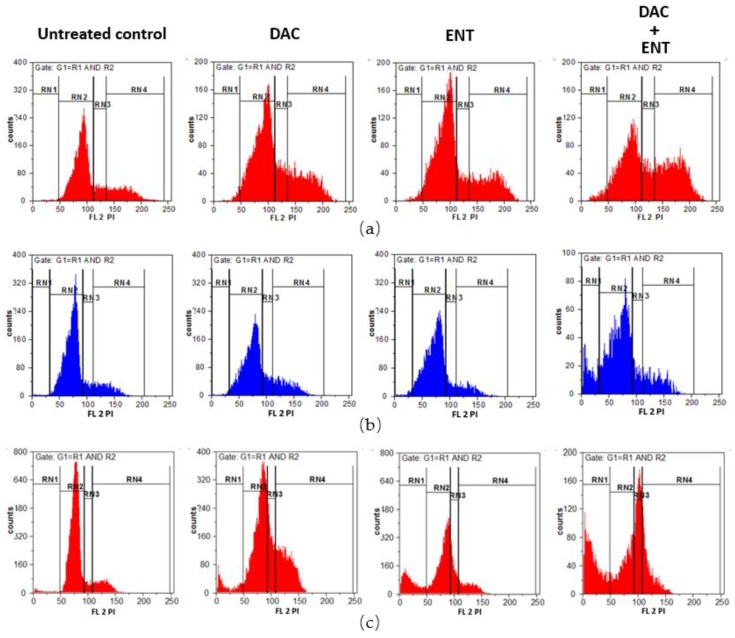

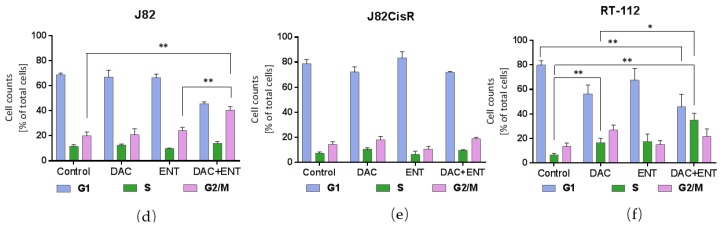
Effect of combination of DAC and ENT on cell cycle progression in J82, J82CisR, and RT-112 cell lines. Cells were incubated with DAC (1 µM in J82 and J82CisR, 0.1 µM in RT-112) or ENT (3.16 µM in J82 and J82CisR, 2 µM in RT-112) or with a combination of DAC and ENT. DMSO was used as a solvent control. RN1, RN2, RN3, and RN4 indicate the cell cycle phases of sub-G1, G1, S, and G2M, respectively. (**a**) Combination treatment led to cell cycle arrest at G2/M phase in J82 cell line. (**b**) Cell cycle distribution of J82CisR cells was not affected by either drug treatment alone or in combination. (**c**) Combination treatment induced cell cycle arrest in S phase in RT-112 cell line. (**d**–**f**) Quantification of the cell cycle distribution after the drug treatments in J82, J82CisR, and RT-112 cell lines. Data shown are the mean ± SD of at least three independent experiments. ** *p* < 0.01, * *p* < 0.05 by *t*-test.

To test whether the combined treatment with DAC and ENT has an effect on the cell cycle distribution of bladder cancer cells, J82, J82CisR, and RT-112 cells were analyzed by flow cytometry with PI staining after treatment of DAC and ENT. In J82 cells, the combination treatment led to cell cycle arrest at the G2/M transition, with 20.0% of cells in G2/M in untreated cells versus 40.5% in DAC plus ENT treated cells (Figure 4a,d). Either drug alone failed to change the cell cycle distribution. In J82CisR, combination treatment only slightly (not significantly) increased the cell population in G2/M phase (Figure 4b,e). In the RT-112 cell line, treatment with DAC alone induced an increase in cells in S- und G2/M phase significantly (16.7% in S, 26.9% in G2/M in DAC-treated versus 6.6% in S and 13.5% in G2/M in untreated cells, *p* < 0.01). Treatment with ENT increased the cell population in S phase to 17.4% but no significant changes were observed in G1 and G2/M phase. Combined treatment with DAC and ENT increased the number of cells in S phase to 34.9% and decreased the number of cells in G1 to 45.7% (Figure 4c,f). Taken together, these data indicate that the combination treatment significantly affects the cell cycle distribution in J82 and RT-112 cell lines but not in J82CisR.

### 2.4. DAC and ENT Alter RNA- and Protein-Expression and Protein Phosphorylation of Genes Involved in the Akt/FoxO Pathway

**Figure 5:**
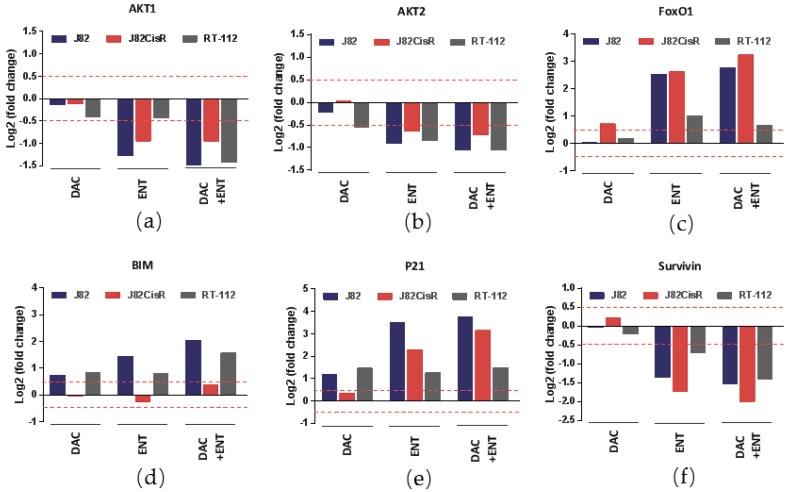
Epigenetic treatments alter gene expression in urothelial bladder cancer cell lines. (**a**–**f**) DAC and ENT resulted in the changes of genes involved in Akt/FoxO pathway. Data shown are RPKM (reads per kilobase per million mapped reads) values normalized from three independent RNA-seq experiments; x axis indicates treatment conditions; y axis indicates log2 fold change of RPKM compared with untreated control.

Differential gene expression was estimated in response to DAC or ENT or the combination of DAC plus ENT using RNA sequencing. Ingenuity pathway analysis revealed that Akt/FoxO signaling was changed remarkably under epigenetic treatment. AKT1 and AKT2, key regulators of cell survival, were downregulated by DAC or ENT to different extents. Treatment with DAC alone had no significant effect on the expression of AKT1 or AKT2. Treatment with ENT reduced expression of AKT1 and AKT2. The combination of DAC with ENT led to an even greater downregulation of AKT1 and AKT2 (Figure 5a,b). Furthermore, forkhead box class O1 (FoxO1), a transcription factor regulated by AKT, was significantly increased by ENT and by the combination treatment DAC plus ENT in J82, J82CisR, and RT-112 (Figure 5c). Finally, a set of target genes of FoxO1 regulating tumor growth and triggering apoptosis (Bim) or inducing cell cycle arrest (p21) were differentially regulated upon treatment with DAC, ENT, or the combination of DAC plus ENT (Figure 5d–f). Expression of BIM and p21 was induced, whereas expression of survivin was downregulated after drug treatment, especially in J82CisR.

**Figure 6:**
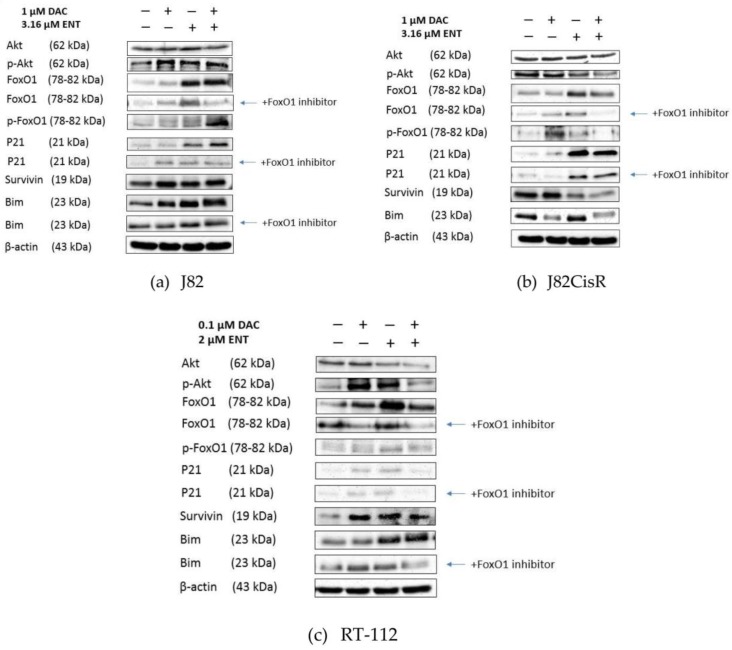
Epigenetic treatments alter protein expression in urothelial bladder cancer cell lines. (**a**–**c**) Western blot analysis of J82, J82CisR, and RT-112 cell lines upon the treatment of DAC and ENT. Cells were treated for 48 h with DAC followed by 48 h treatment with ENT. DMSO was used as a solvent control; the blots shown in (**a**–**c**) are from one representative experiment out of a set of three experiments.

Next, RNA gene expression changes were verified by Western blot analysis. Uncropped Western blots and ratios of integrated densities of proteins of interest and beta-actin from J82, J82CisR, and RT-112 can be found in Figure S3 (J82), Figure S4 (J82CisR), and Figure S5 (RT-112). Consistent with the RNA expression data, FoxO1 was upregulated by either ENT alone or the combination of DAC plus ENT in all three tested cell lines according to Western blots (Figure 6a–c). DAC alone did not (J82CisR) or only slightly increase FoxO1 (J82, RT-112). In accordance with RNA expression, protein levels of Bim, one of the downstream targets of FoxO1, were increased upon treatment with ENT or DAC plus ENT in J82 and RT-112 cells (Figure 6a,b). Bim expression did, however, not increase in J82CisR-neither in RNA nor in protein expression (Figure 5d and Figure 6b). Treatment with ENT or the combination of DAC and ENT significantly increased the protein expression of p21 in J82 and J82CisR, but no significant changes were observed in RT-112 cells. RNA expression of survivin was decreased in all three cell lines upon ENT or DAC plus ENT treatment, whereas a downregulation of survivin protein was only observed in J82CisR (Figure 6b). Next, we analyzed the protein expression of Akt and p-Akt as well as p-FoxO1. While RNA expression suggested a downregulation of Akt in all three cell lines upon ENT and DAC plus ENT treatment, we found a clear reduction of Akt expression in Western blot only in RT-112 cells, whereas in J82 and J82CisR, no treatment-induced changes in Akt expression were found (Figure 6a–c). Interestingly, Akt, the negative upstream regulator of FoxO1, was less phosphorylated (less p-Akt) by treatment with only ENT and even less by treatment with DAC plus ENT in J82CisR, whereas Akt phosphorylation slightly increased in J82 upon treatment with DAC, ENT, or the combination of both. In RT-112, Akt phosphorylation increased upon DAC or ENT treatment but was reduced to the control level upon combination of DAC plus ENT. Since p-Akt can phosphorylate FoxO1 and lead to inactivation of FoxO1, we examined the phosphorylation of FoxO1 (p-FoxO1) in response to DAC, ENT, or combination treatment.

In J82CisR, Akt was less phosphorylated upon DAC plus ENT treatment. As expected, FoxO1 phosphorylation was also reduced in J82CisR by combination treatment (Figure 6b). In J82 cells, increased expression of FoxO1 upon ENT or DAC plus ENT treatment resulted in increased phosphorylation of FoxO1 because p-Akt remained approximately equal over all treatment conditions and was not reduced upon DAC plus ENT treatment (Figure 6a). In RT-112 cells, FoxO1 phosphorylation was rather low but treatment-induced changes correlated with the phosphorylation of Akt (Figure 6c). Notably, the expression of FoxO1 was reduced by the addition of the FoxO1 inhibitor AS1842856 (Figure 6a–c). Furthermore, AS1842856 reduced expression of p21 (J82, J82CisR) and reduced expression of Bim (J82, RT-112). Taken together, the protein expression results demonstrate that DAC and ENT treatment increase FoxO1 expression in urothelial bladder cancer cells. The pathways by which DAC and ENT induce strong synergistic cytotoxicity are, however, different between the three examined bladder cancer cell lines and involve increased p21 expression, increased Bim expression, reduced phosphorylation of Akt, and reduced expression of survivin.

## 3. Discussion

DNMTs have been long recognized for their role in gene silencing by increasing promotor CpG island methylation. This process occurs particularly in cancer cells resulting in the silencing of genes functioning as tumor suppressors [30]. Consequently, targeting DNMT has become a therapeutic approach for anti-cancer treatment as highlighted by the approval of azacytidine and decitabine for treatment of AML or MDS [11]. Similarly, HDACs have become validated drug targets in particular in cancer [31]. HDAC-mediated low acetylation of histones is associated with a condensed chromatin structure resulting in repressed transcription [32]. Because DNA methylation accompanies histone deacetylation in the regulation of gene expression, the combination of DNMTi’s and HDACi’s has been studied in several malignancies including, e.g., acute myelogenous leukemia, non-small cell lung cancer, and colorectal cancer [33,34,35]. In bladder cancer, however, this combination therapy has rarely been investigated. Thus, the aim of the current study was to test the effect of DNMTi’s and HDACi’s in the two muscle-invasive bladder cancer cell lines J82 and RT-112. Since cisplatin is first-line treatment for advanced bladder cancer and cisplatin resistance occurs frequently in bladder cancer patients eventually leading to relapse, we aimed to set up a cellular model of cisplatin resistance in bladder cancer mimicking the clinical development of cisplatin resistance. Thus, the cisplatin-resistant cell line J82CisR was developed according to clinical protocols as previously published by our group for head-neck cancer [27].

In a recent study, we have shown that class I-selective inhibitors like entinostat (ENT) were superior in reversing chemoresistance against cisplatin over pan-HDACi in serous ovarian cancer cell lines [21]. Further, exploratory experiments for the current study revealed that the class I-selective HDACi ENT exhibited a stronger synergistic interaction with the DNMTi DAC than the pan-HDACi panobinostat. This is in agreement with findings that expression levels of class I HDACs are higher in cancer versus non-cancerous cells [36]. The superior effect of ENT in combination with DAC compared with other classes of HDACi’s may also be explained by the fact that class I HDACs are mainly found in the nucleus and regulate the acetylation status of histones [34].

The main finding of our study is that the DNMTi DAC and the HDACi ENT act synergistically in reducing the viability of urothelial bladder cancer cells by inducing apoptosis and cell cycle arrest (Figure 3 and Figure 4). Importantly, the results from the non-cancerous urothelial bladder cell line HBLAK indicated selectivity of the combination treatment DAC plus ENT for bladder cancer over non-cancer cells (5- to 23-fold for the cell lines J82, J82CisR, and RT-112). Treatment with DAC or ENT alone shows only moderate cytotoxicity and almost no apoptosis induction (Table 1 and Figure 3). Combination of both compounds, however, induced strong apoptosis as seen by sub G1 increase mediated by the activation of caspases 3/7 (Figure 3). These findings are in accordance with previous studies that investigated the effects of DNMTi and HDACi in other malignancies such as acute myelogenous leukemia, non-small cell lung cancer, or colorectal cancer [33,34]. Notably, in contrast to our results from ovarian and head-neck cancers, neither DAC nor ENT nor the combination of DAC plus ENT could completely reverse cisplatin resistance in the bladder cancer cell line J82CisR [19,21] (Figure S2). Gene expression experiments of J82 and J82CisR did not reveal major changes of DNA damage response (DDR) genes, nor did they unravel the exact mechanism of resistance against cisplatin, as typical cisplatin resistance genes such as DNA excision repair gene ERCC-1, the copper transporter CTR1, and multidrug resistance-associated protein 2 ABCC2 remained unchanged. However, rather than further examining the resistance mechanism of J82CisR, we aimed at exploring the novel therapeutic combination of DAC (approved drug) and ENT (late-stage clinical phase 3) for the treatment of chemoresistant bladder cancer. Mechanistically, we found an upregulation of the transcription factor FoxO1 in all three examined bladder cancer cell lines upon epigenetic treatment with DAC, ENT, or the combination of DAC plus ENT. FoxO1 is a transcription factor negatively regulated through phosphorylation by activated Akt (p-Akt) [37]. Multiple downstream targets of FoxO1 play an essential role in controlling cell survival and proliferation, such as the proapoptotic protein Bim, the cell cycle regulator p21, or the survival protein survivin [38], which was confirmed in our study by using the FoxO1 inhibitor AS1842856 (Figure 6a–c).

FoxO1 was upregulated in all three cell lines upon epigenetic treatment albeit to different extents (Figure 6a–c). However, the mechanisms of FoxO1 activation are distinct in the various cell lines upon the treatment with DAC, ENT, or DAC plus ENT. In J82, upregulation of FoxO1 was observed independent of increased p-Akt levels upon epigenetic treatment (Figure 6a). p-FoxO1 increased following increased phosphorylation of Akt after the combination treatment DAC plus ENT. In contrast, in J82CisR cells, the combination of DAC plus ENT reduced the phosphorylation of Akt, subsequently resulting in reduced p-FoxO1 and thus in activation of FoxO1. In RT-112 cells, an increase in FoxO1 expression did not correlate with phosphorylated Akt, similar to the results in J82. We thus conclude that epigenetic modulation with DAC and ENT induced increased expression of FoxO1 independent of the status of p-Akt in J82 and RT-112 cells whereas in J82CisR, increased FoxO1 correlates with reduced phosphorylation of Akt. These findings may have clinical significance, since high FoxO1 expression levels result in increased relapse-free survival of bladder cancer patients as shown by analysis of publicly available bladder cancer expression data (Figure S6). Further, in J82CisR, reduced phosphorylation of Akt also correlated with reduced expression of survivin after combination of DAC plus ENT (Figure 6b), eventually leading to increased apoptosis. These data are also in accordance with literature data [39] and confirm the distinct mechanisms of the cytotoxicity induced by epigenetic agents in J82, RT-112, and J82CisR cells. Although pharmacological modulation by the FoxO1 inhibitor AS1842856 (Figure 6) should ideally be confirmed by molecular biology studies such as siRNA knockdown experiments, besides clear convincing results obtained with the FoxO1 inhibitor AS1842856 (Figure 6), the clinical importance of FoxO1 expression was shown by analysis of publicly available bladder cancer expression data presented in Figure S6. In the next step, synergy between DAC and ENT needs to be investigated in a larger number of bladder cancer cell lines, in particular in cisplatin-resistant cell lines. Criteria for inclusion of patients in prospective clinical trials may be low FoxO1 expression in tumor specimens and resistance to the first-line therapy cisplatin.

## 4. Materials and Methods

### 4.1. Materials

Dulbecco’s Modified Eagle Medium (DMEM) was obtained from Gibco (Darmstadt, Germany). Penicillin/streptomycin (pen/strep) (10,000 U/mL; 10 mg/mL) and trypsin-EDTA (0.05% Trypsin, 0.02% EDTA in Phosphate Buffer Saline) were purchased from PAN Biotech (Aidenbach, Germany). Cisplatin was ordered from Sigma (Sigma-Aldrich, Steinheim, Germany). Decitabine, Entinostat, and FoxO1 inhibitor AS1842856 (Selleckchem, Houston, TX, USA) were prepared at 10 mM in DMSO. Cisplatin was dissolved and subsequently diluted in 0.9% saline. The caspase inhibitor Q-VD-OPh (Sigma-Aldrich, Steinheim, Germany) was dissolved in DMSO at 10 mM and diluted in DMEM. Hoechst 33342 (Sigma-Aldrich, Steinheim, Germany) was dissolved in distilled water at 10 mg/mL. 3-(4,5-dimethylthiazol-2-yl)-2,5-diphenyltetrazolium bromide (MTT) was dissolved in PBS at a concentration of 5 mg/mL and obtained from Serva (Heidelberg, Germany). Propidium iodide was obtained from Santa Cruz Biotechnology (Heidelberg, Germany). Triton X-100 was purchased from AppliChem (Darmstadt, Germany). 0.9% NaCl, obtained from Fresenius Kabi (Bad Homburg, Germany) and supplemented with 0.01% sodium azide, was used as sheath fluid for flow cytometry analysis. The CellEvent Caspase-3/7 green detection reagent was purchased from Invitrogen (Carlsbad, CA, USA). HRP-conjugated secondary antibodies used in Western blotting were ordered from R&D Systems (Wiesbaden, Germany).

### 4.2. Cell Culture

The urothelial bladder cancer cell lines J82, RT-112 and the normal human bladder cell line HBLAK were kindly provided by Prof. Wolfgang A. Schulz (Department of Urology, Medical Faculty, Heinrich Heine University). The J82 cisplatin-resistant (CisR) cell line was generated by exposing the parental cell line to weekly treatment with cisplatin at an IC_50_ concentration over a period of 34 weeks as previously described [27]. The cells were cultured in DMEM supplemented with 10% fetal bovine serum, 120 µg/mL streptomycin, and 120 U/mL penicillin. All cells were cultured at 37°C in a humidified atmosphere containing 5% CO_2_. When cells reached a confluence of 80–90%, they were washed with 1× PBS and treated with trypsin-EDTA before use for the respective experiments.

### 4.3. Cell Viability Assay

The MTT assay was used to test the cell viability and was performed as previously described [40]. Briefly, cells were seeded into 96-well plates (Sarstedt, Nümbrecht, Germany) and incubated overnight. Then, cells were treated with increasing concentrations of test agent. After a certain incubation period, 25 µL of MTT solution (5 mg/mL) was added into each well. After 15 min incubation, the mixture of medium and MTT solution was removed, and 75 µL of DMSO was added to dissolve formazan crystals. Absorbance of each well was measured at 544 nm (test wavelength) and 690 nm (background) using the BMG FLUOstar (BMG Labtechnologies Offenburg, Germany). Background was subtracted from the absorbance of each well.

### 4.4. Synergistic Study

To determine the synergism of DAC and ENT, the rates of cell growth inhibition were obtained and calculated from MTT assays. The combination indexes (CIs) were calculated using Compusyn software version 1.0 (ComboSyn, Inc., Paramus, NJ, USA) based on the Chou-Talalay method. The synergism, additive effect, and antagonism are defined by CI < 1, CI = 1, and CI > 1, respectively [41].

### 4.5. Apoptosis Assay

The apoptosis assay was performed as previously described [42]. Briefly, cells were seeded in 24-well plates and incubated overnight. Then the cells were exposed to DAC for 48 h followed by additional 48 h treatment with ENT. The caspase inhibitor Q-VD-OPh was added 1 h prior to the compound treatment (20 µM). To lyse the cells, the plate was centrifuged and the supernatant was removed carefully. To each well, 500 µL of hypotonic lysis buffer (sodium citrate tribasic dihydrate 0.1%, Triton X-100 0.1% (*v*/*v*), PI 100 µg/mL) was added, and the 24-well plate was stored at 4 °C in the dark overnight. The counts in sub-G_1_ of the cell nuclei were detected by flow cytometry (CyFlow^®^ space of Partec, Münster, Germany).

### 4.6. Cell Cycle Analysis

For cell cycle analysis, the cells were plated in 6-well plates 1 day before the drug treatment. The cells were treated with DAC for 48 h and an additional 48 h with ENT either alone or combined with DAC. The cells were then collected and washed with PBS and fixed in 70% ice cold ethanol at –20 °C for 24 h. The fixed cells were washed with cold PBS and incubated in staining solution containing 0.1% (*v/v*) Triton X-100, 200 µg/mL DNAse-free RNAse A (Fermentas/Thermo Fisher Scientific, Waltham, MA, USA), and 20 µg/mL propidium iodide for 15 min at 37 °C (protected from light). The DNA content was measured by flow cytometry and the doublet discrimination mode was used.

### 4.7. Caspase 3/7 Activation

The detection of caspase 3/7 activity after DAC and ENT treatment was performed using high-content analysis (HCA). The cells were treated in the same manner as described in apoptosis assay and cell cycle analysis. After drug incubation, the medium was aspirated, and the cells were labeled with the mixture of CellEvent^TM^ Caspase-3/7 green detection reagent and Hoechst 33342. After 30 min incubation, images were acquired by ArrayScan XTI Live High Content Platform (Thermo Fisher Scientific Inc., Waltham, MA, USA) using excitation filters at 386 and 485 nm for Hoechst 33342 and Caspase-3/7 green detection reagent, respectively. The percentage of activated caspase 3/7 cells was calculated using HCS Studio Cellomics Scan (Thermo Fisher Scientific Inc.).

### 4.8. Total RNA Extraction and RNA-seq Analysis

Total RNA was isolated using RNeasy Mini Kit (QIAGEN, Hilden, Germany) according to the manufacturer’s instruction. The total RNA samples used for transcriptome analyses were quantified (Qubit RNA HS Assay, Thermo Fisher Scientific) and quality-measured by capillary electrophoresis using the Fragment Analyzer and the ‘Total RNA Standard Sensitivity Assay’ (Agilent Technologies, Inc. Santa Clara, CA, USA). All samples in this study showed high RNA quality numbers (RQN; mean = 9.9). The library preparation was performed according to the manufacturer’s protocol using the Illumina^®^ ‘TruSeq Stranded mRNA Library Prep Kit’. Briefly, 300 ng total RNA were used for mRNA capturing, fragmentation, the synthesis of cDNA, adapter ligation, and library amplification. Bead purified libraries were normalized and finally sequenced on the HiSeq 3000/4000 system (Illumina Inc. San Diego, CA, USA) with a read setup of 1 × 150 bp. The bcl2fastq tool was used to convert the bcl files to fastq files as well for adapter trimming and demultiplexing.

### 4.9. Analysis of RNA-Seq Data

Data analyses on fastq files were conducted with CLC Genomics Workbench (version 10.1.1, QIAGEN, Venlo. NL). The reads of all probes were adapter trimmed (Illumina TruSeq) and quality trimmed (using the default parameters: bases below Q13 were trimmed from the end of the reads, with a maximum of two ambiguous nucleotides). Mapping was done against the Homo sapiens (GRCh38) (Mai 25, 2017) genome sequence. After grouping of samples (three biological replicates each) according to their respective experimental condition, multi-group comparisons were made and statistically determined using the Empirical Analysis of DGE (version 1.1, cutoff = 5). The resulting *p* values were corrected for multiple testing by FDR and Bonferroni-correction. A *p* value of ≤0.05 was considered significant. Data were further evaluated with the Ingenuity-Pathway analysis software (Qiagen Inc. 2016).

### 4.10. Western Blot Analysis

Total protein extraction and Western blot analysis were performed as previously described with minor modification [43]. Briefly, cells were lysed with RIPA buffer (150 mM NaCl, 1% Triton X-100, 0.5% Na-desoxycholate, 0.1% SDS, 2 mM EDTA, 50 mM Tris-HCl pH 8.0) containing Pierce protease and phosphatase inhibitor mini tablets (Thermo Scientific, Rockford, IL, USA) and boiled at 95 °C with 2× Laemmli-buffer containing β-mercaptoethanol for 5 min. The concentration of the total protein was determined by Pierce BCA protein assay (Thermo Scientific, Rockford, IL, USA). Equal amount of proteins was loaded onto SDS-PAGE for separation. Proteins were transferred on polyvinylidene difluoride membrane (Merck Millipore, Darmstadt, Germany) by semi-dry blotting for 1 h. Blots were blocked in either Tris Buffered Saline-0.1% Tween 20 (TBST) 3% milk or TBST-3% bovine serum albumin (AppliChem, Darmstadt, Germany) for 1 h. Primary antibodies were incubated at 4 °C overnight. For information on the primary antibodies, we refer to the Supplementary Information. Blots were washed twice with TBST and once with TBS followed by the HRP-conjugated secondary antibody incubation for 1 h. Blots were then washed again twice with TBST and once with TBS and detected using the Western Blotting Luminol Reagent (Santa Cruz Biotechnologies, Heidelberg, Germany) and the INTAS Science Imaging Instrument (GeliX Imager, Göttingen, Germany).

### 4.11. Statistical Analysis 

The statistical analysis was carried out with GraphPad Prism 7.0 (GraphPad Software Inc., San Diego, CA, USA). The two-tailed Student’s *t*-test or ANOVA was used, and *p*-value less than 0.05 was considered as significant. Concentration-effect curves were then generated by nonlinear regression curve fitting using the 4-parameter logistic equation with variable hill slope. The IC_50_ values are the concentration of the cytotoxic agent that led to a decrease of 50% of the recorded signal. The pIC_50_ values are −log IC_50_. Assays were performed at least in three independent experiments each carried out in triplicates. Values shown are mean ± SEM.

## 5. Conclusions

In conclusion, our study demonstrated that the combination treatment of DAC plus ENT is cancer-selective and highly synergistic in inhibiting the proliferation of urothelial bladder cancer cells via induction of apoptosis and caspase 3/7 activation. Mechanistically, epigenetic treatment resulted in the increased expression of FoxO1 which then induced cell line-dependent further pathways, such as increased expression of Bim or p21 or reduced expression of survivin. Our findings provide evidence that a combination of the approved drug decitabine and the late-stage trial drug (phase 3) entinostat is a promising therapeutic strategy for bladder cancer, in particular for patients resistant to the first-line therapy cisplatin.

## Figures and Tables

**Table 1:** Summary of the IC_50_ and pIC_50_ [-log IC_50_] values of DAC and ENT in J82, J82CisR, and RT-112 cells (72 h incubation).

Cell Lines	DAC		ENT	
	IC_50_ [µM]	pIC_50_ ± SEM	IC_50_ [µM]	pIC_50_ ± SEM
J82	30.5	4.52 ± 0.08	14.3	4.84 ± 0.06
J82CisR	28.2	4.55 ± 0.18	15.6	4.81 ± 0.06
RT-112	0.22	6.65 ± 0.09	3.41	5.47 ± 0.11

Data shown are the mean of three independent experiments ± SEM.

## Supplementary Materials

The following are available online at https://www.mdpi.com/2072-6694/12/2/337/s1, Figure S1: Evaluation of the cytotoxic effects of DAC and ENT in bladder cancer cell lines, Figure S2: Combination treatment of DAC or ENT with cisplatin, Figure S3: Uncropped Western blots and ratios of integrated densities of proteins of interest and beta-actin from J82 cells, Figure S4: Uncropped Western blots and ratios of integrated densities of proteins of interest and beta-actin from J82CisR cells, Figure S5: Uncropped Western blots and ratios of integrated densities of proteins of interest and beta-actin from RT-112 cells, Figure S6: Kaplan-Meier plot showing relapse free survival of 187 bladder cancer patients with low or high FoxO1 expression levels.
