# Supplementary material for: Combination of Decitabine and Entinostat Synergistically Inhibits Urothelial Bladder Cancer Cells via Activation of FoxO1
Source: Cancers (Basel). 2020 Feb 3;12(2):337. doi: 10.3390/cancers12020337 (PMC7073167; doi:10.3390/cancers12020337)
Supplement: Supplementary file 1 [file cancers-12-00337-s001.docx]

| Supplementary Materials: Combination of Decitabine and Entinostat Synergistically Inhibits Urothelial Bladder Cancer Cells via Activation of FoxO1  Chenyin Wang, Alexandra Hamacher, Patrick Petzsch, Karl Köhrer, Günter Niegisch, Michèle J. Hoffmann, Wolfgang A. Schulz and Matthias U. Kassack    (a) |
| --- |
|   (b) |

**Figure S1.** Evaluation of the cytotoxic effects of DAC and ENT in bladder cancer cell lines. (**a**) Determination of IC_50_ values of DAC with 48 plus 72 h treatment in J82, J82CisR, and RT-112 cell lines. (**b**) Determination of IC_50_ values of ENT with 72 h treatment in J82, J82CisR, and RT-112 cell lines.


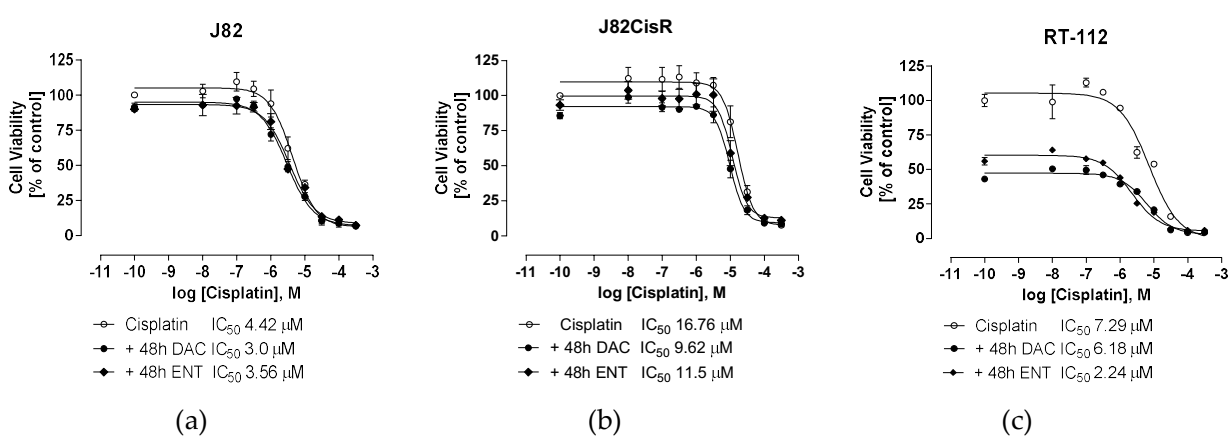


**Figure S2.** Combination treatment of DAC or ENT with cisplatin. (**a**) 48 h pre-incubation of DAC or ENT following 72 h treatment of cisplatin in J82 cell line. (**b**) 48 h pre-incubation of DAC or ENT following 72 h treatment of cisplatin in J82CisR cell line. (**c**) 48 h pre-incubation of DAC or ENT following 72 h treatment of cisplatin in RT-112 cell line.


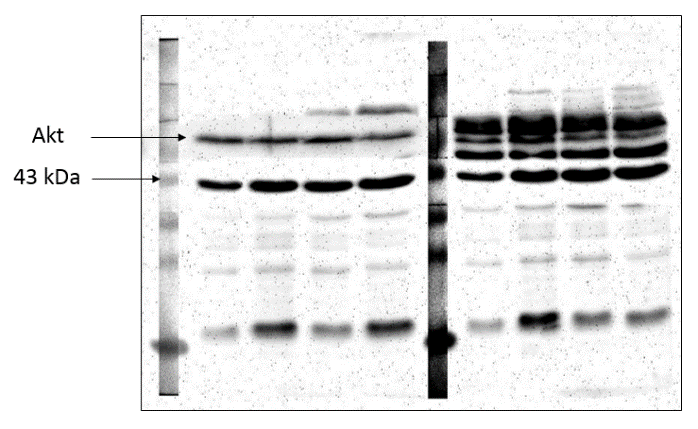


(a)


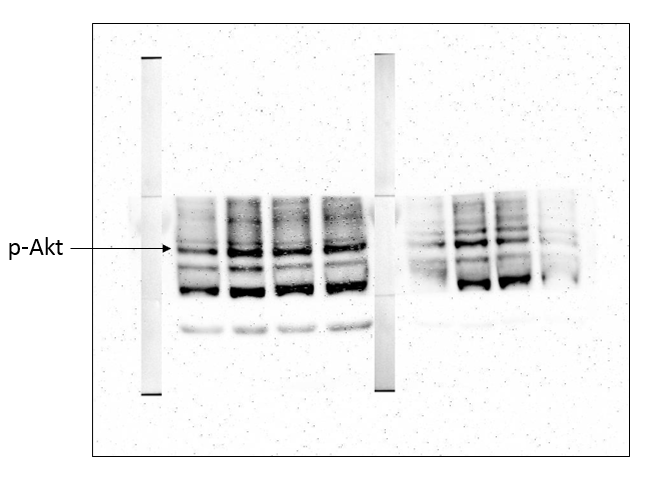


(b)


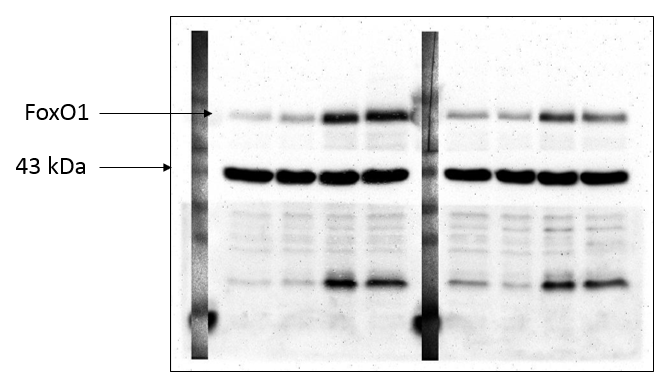


(c)


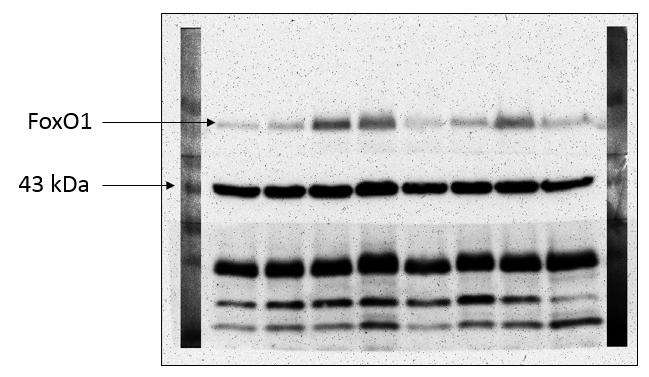


(d)


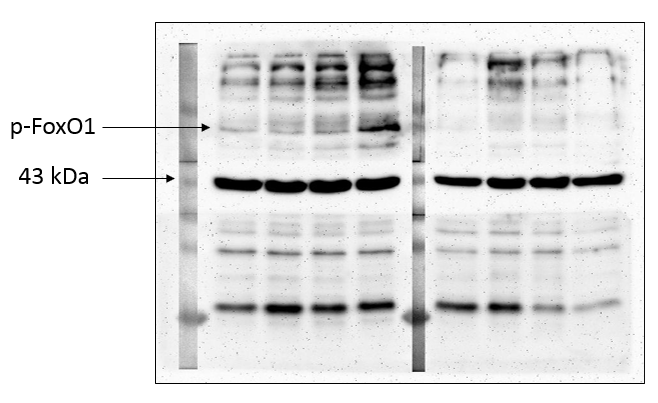


(e)


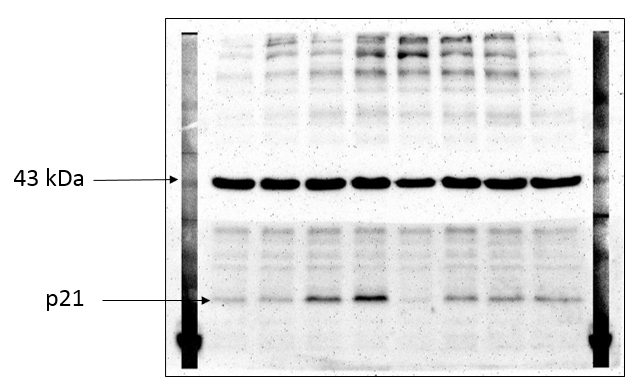


(f)


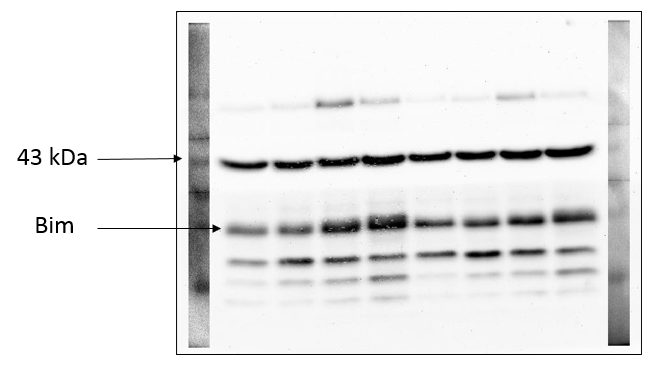


(g)


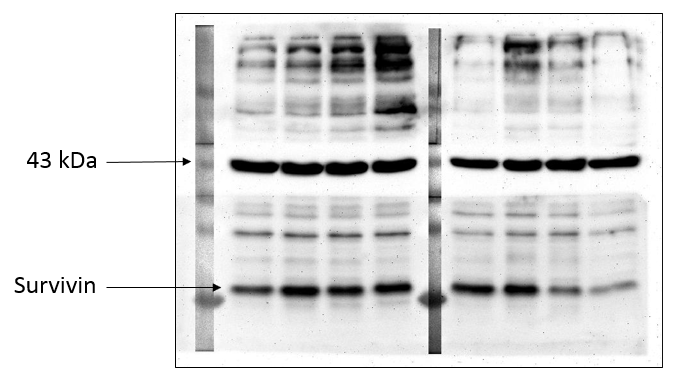


(h)

(i)

**Figure S3.** Uncropped western blots and ratios of integrated densities of proteins of interest and beta-actin from J82 cells. Lanes correspond to Control (untreated), treatment with DAC, ENT, or DAC + ENT. Bands labeled with 43 kDa correspond to beta actin. (**a**) Expression of Akt. (**b**) Expression of p-Akt. (**c**) Expression of FoxO1. (**d**) Expression of FoxO1 in presence of FoxO1 inhibitor. (**e**) Expression of p-FoxO1. (**f**) Left 4 lanes: expression of p21. Right 4 lanes: expression of p21 in presence of FoxO1 inhibitor. (**g**) Left 4 lanes: expression of Bim. Right 4 lanes: expression of Bim in presence of FoxO1 inhibitor. (**h**) Expression of survivin. (**i**) Ratios of integrated densities of proteins of interest and beta-actin from J82 cells.


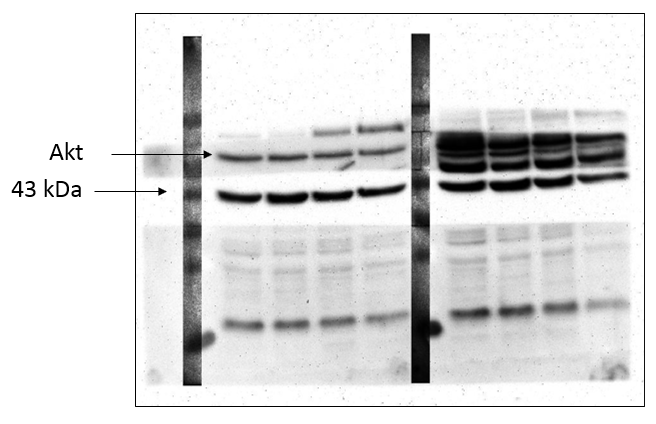


(a)


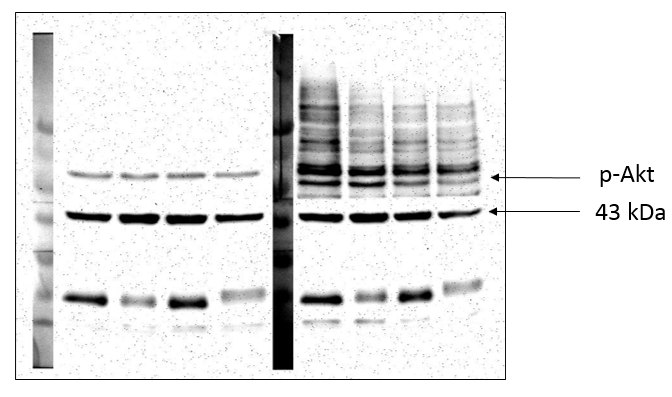


(b)


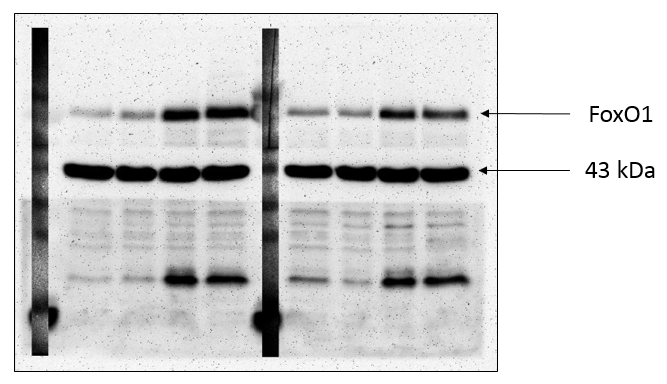


(c)


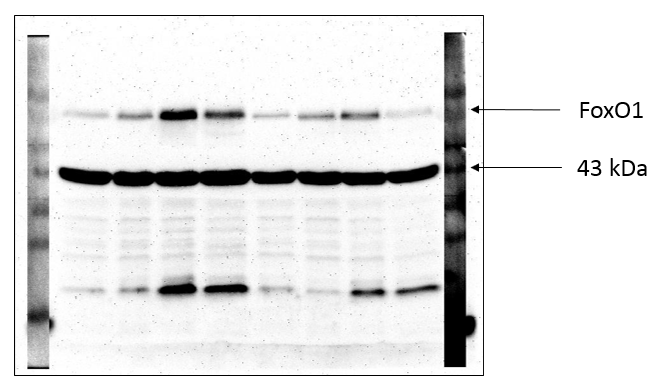


(d)


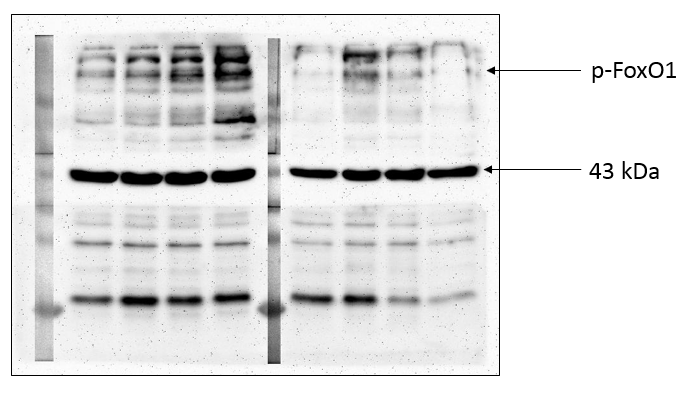


(e)


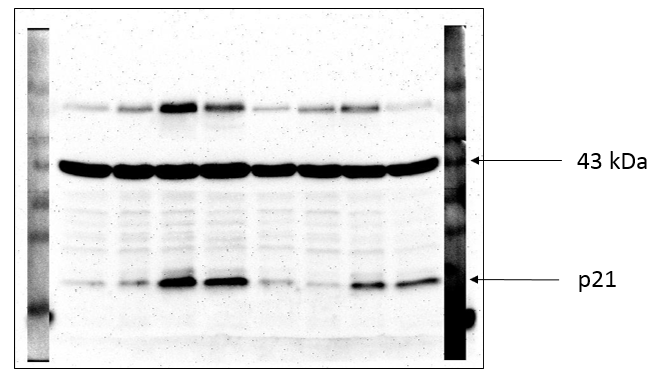


(f)


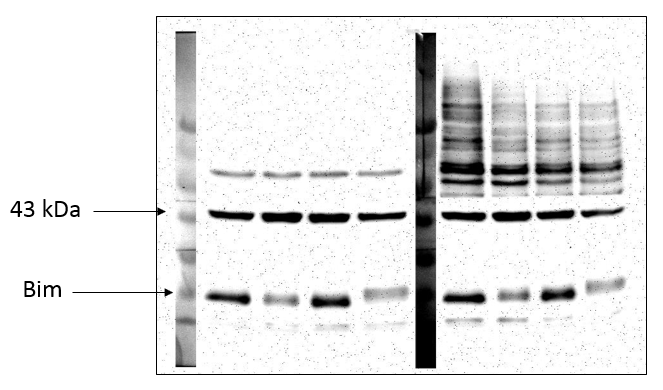


(g)


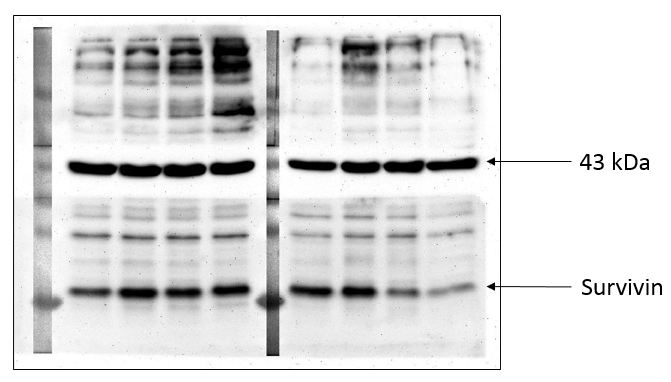


(h)

(i)

**Figure S4.** Uncropped western blots and ratios of integrated densities of proteins of interest and beta-actin from J82CisR cells. Lanes correspond to Control (untreated), treatment with DAC, ENT, or DAC + ENT. Bands labeled with 43 kDa correspond to beta actin. (**a**) Expression of Akt. (**b**) Expression of p-Akt. (**c**) Expression of FoxO1. (**d**) Expression of FoxO1 in presence of FoxO1 inhibitor. (**e**) Expression of p-FoxO1. (**f**) Left 4 lanes: expression of p21. Right 4 lanes: expression of p21 in presence of FoxO1 inhibitor. (**g**) Expression of Bim. (**h**) Expression of survivin. (**i**) Ratios of integrated densities of proteins of interest and beta-actin from J82CisR cells.


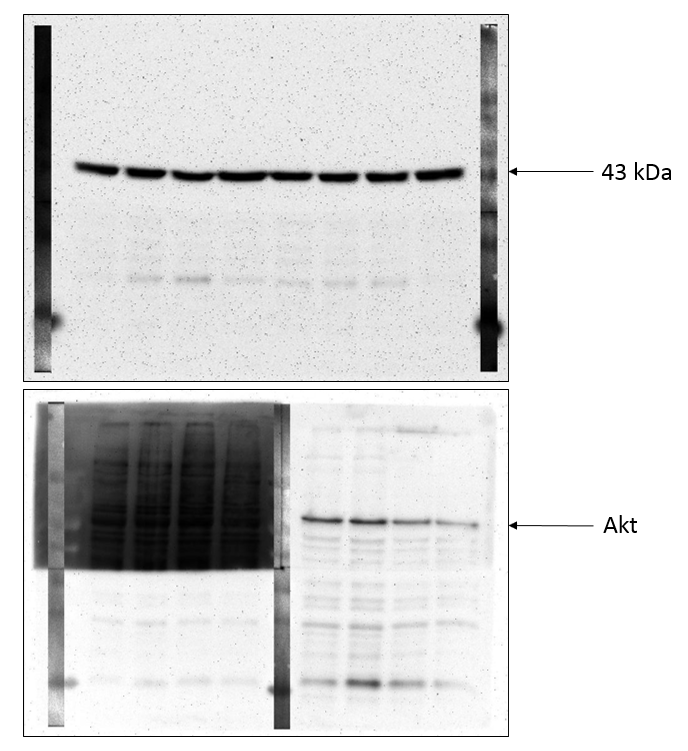


(a)


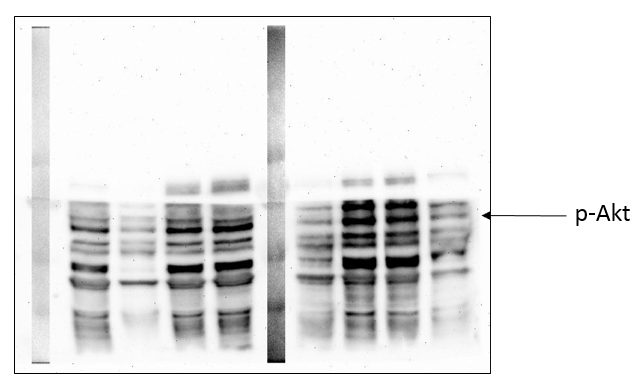


(b)


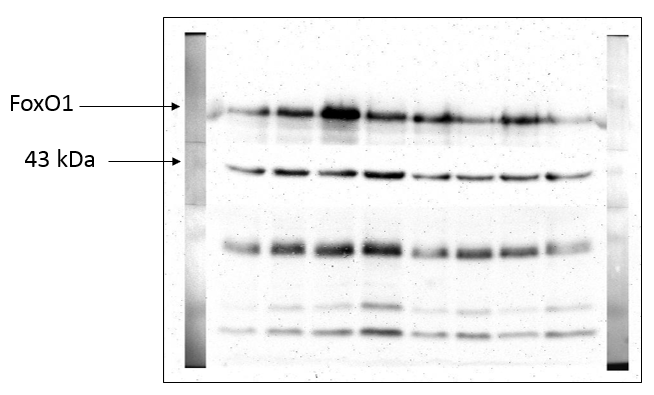


(c)


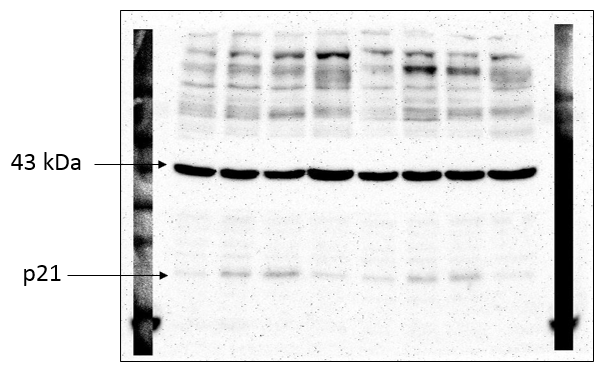


(d)


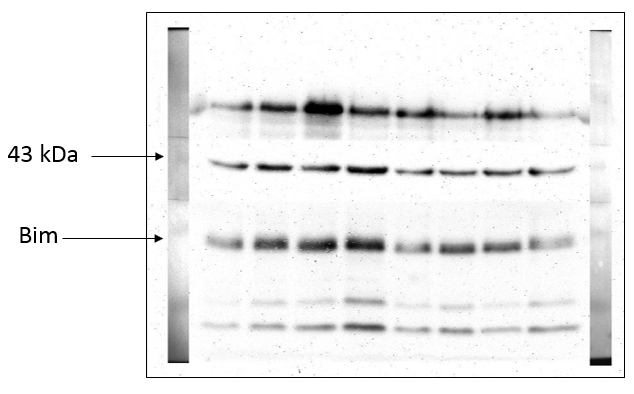


(e)


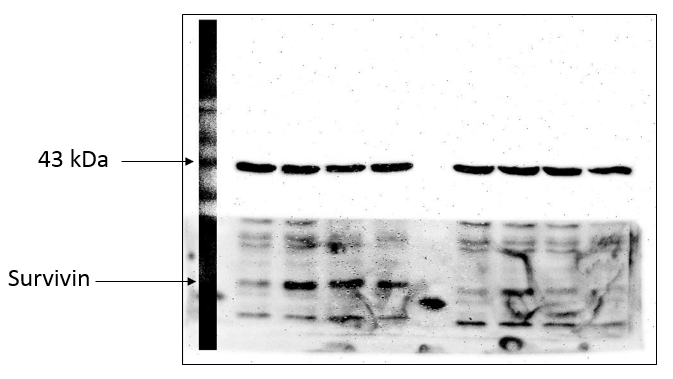


(f)

(g)

**Figure S5.** Uncropped western blots and ratios of integrated densities of proteins of interest and beta-actin from RT-112 cells. Lanes correspond to Control (untreated), treatment with DAC, ENT, or DAC + ENT. Bands labeled with 43 kDa correspond to beta actin. (**a**) Expression of Akt. (**b**) Expression of p-Akt. (**c**) Left 4 lanes: expression of FoxO1. Right 4 lanes: expression of FoxO1 in presence of FoxO1 inhibitor. (**d**) Left 4 lanes: expression of p21. Right 4 lanes: expression of p21 in presence of FoxO1 inhibitor. (**e**) Left 4 lanes: expression of Bim. Right 4 lanes: expression of Bim in presence of FoxO1 inhibitor. (**f**) Expression of survivin. (**g**) Ratios of integrated densities of proteins of interest and beta-actin from RT-112 cells.


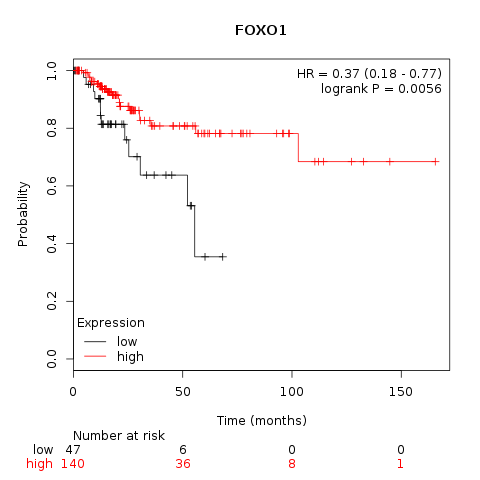


**Figure S6.** Kaplan Meier plot showing relapse free survival of 187 bladder cancer patients with low or high FoxO1 expression levels. Data were extracted from publicly available database KM plotter (https://kmplot.com) described in Nagy A, Lánczky A, Menyhárt O, Győrffy B. Validation of miRNA prognostic power in hepatocellular carcinoma using expression data of independent datasets, Scientific Reports, 2018;8:9227.

| 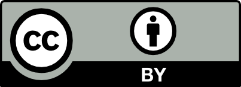 | © 2020 by the authors. Licensee MDPI, Basel, Switzerland. This article is an open access article distributed under the terms and conditions of the Creative Commons Attribution (CC BY) license (http://creativecommons.org/licenses/by/4.0/). |
| --- | --- |
